# Variations in the Terminal Branches of the Brachial Plexus in the Axillary Fossa

**DOI:** 10.1155/bmri/5547451

**Published:** 2025-01-18

**Authors:** Elena Bozhikova, Stoyan Novakov, Tsvetanka Petleshkova, Zdravka Harizanova, Nikolay Uzunov

**Affiliations:** ^1^Department of Biomedical Sciences, Mercer University School of Medicine, Columbus, Georgia, USA; ^2^Department of Anatomy, Histology and Embryology, Medical University–Plovdiv, Plovdiv, Bulgaria; ^3^Department of Maxillofacial Surgery, University Hospital “Medika”, Ruse, Bulgaria

**Keywords:** axillary nerve, brachial plexus, brachial plexus variations, communicating branches, musculocutaneous nerve, radial nerve

## Abstract

**Background:** The brachial plexus is the primary nerve source for the upper limb. Variations in its anatomy can alter the nerve supply to the upper extremity. Such deviations are clinically important, as they can change the symptomatology of various pathologic conditions, leading to misdiagnosis, inadequate treatment, and surgical failures as a consequence.

**Materials and Methods:** The upper limbs of 16 human cadavers (32 extremities) were dissected at the Department of Anatomy, Histology and Embryology, Medical University–Plovdiv, Bulgaria. Eight cadavers were male and eight female, with age ranging 40–95 years (mean age: 72.63 years).

**Results:** Three variations (18.8%) were identified unilaterally, exclusively in male cadavers; however, no statistically significant sex-based distribution was observed. The first variation involved a communicating branch between the musculocutaneous nerve and median nerve. The second was a high bifurcation of the radial nerve in the axillary fossa into two divisions. The third one was a communicating branch between the axillary nerve and the radial nerve, forming a loop beneath the subscapular artery.

**Conclusion:** Our study identified greater variability in the branches of the posterior cord compared to the existing literature. These variations must be taken under clinical consideration to prevent diagnostic, therapeutic, and surgical errors.

## 1. Introduction

The brachial plexus (BP) serves as the primary neural source for the upper limb. It is composed of roots, trunks, divisions, cords, and terminal branches. The cords of the BP are situated in the axillary fossa around the axillary artery (AA). The lateral cord (LC) is formed by the union of the anterior divisions (ADs) of the upper and middle trunk. The medial cord (MC) is derived from the AD of the inferior trunk, while the posterior cord (PC) arises from the convergence of the posterior divisions of all three trunks. The branches, originating from the LC, are the lateral pectoral nerve, the musculocutaneous nerve (MCN), and the lateral root (LR) of the median nerve (MN). The branches from the MC are the medial pectoral nerve, the medial cutaneous nerve of the arm and forearm, the ulnar nerve (UN), and the medial root (MR) of the MN. The PC gives rise to the subscapular nerves, the thoracodorsal nerve (TDN), the axillary nerve (AN), and the radial nerve (RN) [1].

Variations of the BP are well documented. Their high prevalence, clinical importance, and the susceptibility of the BP to injury have generated significant research interest in the deviations of the plexus, particularly regarding their implications for diagnostic and surgical procedures [2, 3]. BP injuries can occur after trauma and compression, including car accidents, gunshot wounds and stab injuries, complicated births, open fractures of the shoulder girdle, and ablative surgery. The evaluation and treatment of such injuries are often complicated by the anatomical variability of the BP [3].

A comprehensive understanding of the anatomical variations of the BP is essential for the development of new surgical reconstructive procedures, the improvement of regional anesthesia techniques, and the enhancement of radiological methods for the evaluation of the peripheral nerves. Furthermore, such knowledge is fundamental to optimizing treatment approaches, including innovative nerve transfer techniques and neurotization procedures [3–5].

The variations of the BP are of critical importance in understanding the pathophysiology of nerve dysfunctions, interpreting motor and sensory deficits in the upper limb, and planning effective neurological and surgical interventions. They are particularly significant for conducting neurophysiological assessments, accurately interpreting clinical manifestations, and mitigating the risk of iatrogenic nerve damage during surgical procedures [6–12].

The variations of the BP must be carefully considered during various diagnostic evaluations, including posttraumatic assessments, clinical neuropathy diagnosis, and exploratory interventions for peripheral nerve repairs [3, 10, 11, 13, 14]. Such variations can significantly alter the sensory and motor innervation of the upper limb, leading to atypical clinical presentations and increasing the risk of diagnostic inaccuracies [3, 15–17]. The deviations of the BP are associated with the development of various pathological conditions, such as thoracic outlet syndrome, entrapment syndromes, neuralgias, or paralysis due to altered relationships between the nerve and the adjacent musculature [2, 3, 8, 12, 18, 19]. Unawareness of such variations may result in failure of regional anesthesia, ineffective treatment of the BP injuries and tumors, and intra- and postoperative complications such as nerve or vascular injuries [3, 4, 11, 20]. The variations of the BP are also particularly vulnerable to injuries during interventions involving the axilla or upper arm, such as nerve anesthesia, repair of shoulder dislocations, fixation of humeral fractures, resection of axillary tumors, lymph node biopsy, axillary lymphadenectomy, or radical mastectomy [2, 14, 18, 21]. Enhanced understanding and recognition of BP variations are essential to improve the safety and efficacy of diagnostic, therapeutic, and surgical procedures.

Although numerous publications have addressed the variations of the BP, many of these studies are limited to case reports [3]. Given the clinical significance of BP variations, continued research is essential to deepen the understanding of their implications and to improve diagnostic, therapeutic, and surgical outcomes for individuals with BP injuries. Due to the lack of comprehensive data on BP variations in the Bulgarian population, the aim of the present study was to investigate the prevalence and types of variations in the terminal branches of the BP in the axillary fossa on Bulgarian cadavers.

## 2. Materials and Methods

The upper limbs of 16 human cadavers (32 plexuses) were dissected in the Department of Anatomy, Histology and Embryology at the Medical University–Plovdiv, Bulgaria. A detailed dissection of the axillary fossa and arm was performed. The study included eight male and eight female cadavers, with ages ranging from 40 to 95 years (mean age: 72.63 years). All cadavers were preserved in a solution containing 96% denatured alcohol, 36% formaldehyde, glycerol, carbolic acid, and water. The inclusion criteria required the BP and its terminal branches to be intact and free of pathological changes.

The dissection of the cadavers was conducted in accordance with the protocols outlined in Grant's Dissector, 16th edition [22]. To expose the contents of the axillary fossa and arm, multiple precise skin incisions were performed. We used a midline incision along the sternum and horizontal incisions from the jugular notch along the clavicle to the acromion, from the midpoint of the sternal manubrium to the midaxillary line, and from the xiphisternal junction to the midaxillary line. A transverse incision was placed midway between the incision along the clavicle and the incision extending from the middle of the sternum. Another incision extended from the xiphisternal junction along the costal margin to the midaxillary line. For the upper limb, a longitudinal incision was made from the acromion along the lateral side of the arm and another along the medial side, both extending approximately 1 cm below the elbow. These longitudinal incisions were connected by a circular horizontal incision distal to the elbow and an additional horizontal incision midway along the arm. A final incision was performed along the midaxillary line.

The skin and superficial fascia of the pectoral region, axillary fossa, and arm were carefully excised, with preservation of the superficial veins and cutaneous nerves. The fascia over the deltoid and pectoralis major muscles was removed. Using blunt dissection, the pectoralis major muscle was separated from the pectoralis minor. The clavicular head of the pectoralis major was cut just inferior to the clavicle, while the sternal head was incised approximately 1 cm lateral to its sternal attachment. The muscle was reflected laterally, preserving its humeral attachment. The clavipectoral fascia was exposed and carefully removed from the anterior surface of the pectoralis minor. Subsequently, the muscle was detached from the ribs and reflected superiorly. The upper limb was abducted laterally, and the axillary fat, axillary sheath, brachial fascia, and axillary lymph nodes were excised. The arm muscles were separated using blunt dissection to avoid damage to underlying structures.

The axillary vessels and cords of the BP were identified, the surrounding connective tissue was removed, and the structures were isolated and separated. The branches of the AA were carefully cleaned. Using the coracobrachialis muscle (CBM) as a landmark, the MCN and the LR of the MN were identified. The LR was followed distally to identify and clean the MN. Superiorly, the MR of the MN was traced to expose the MC of the BP, and its branches were subsequently identified and cleaned.

In each cadaver, direct observation of the BP and its branches was conducted, and any observed variations were documented and photographed. All measurements were made using an electronic caliper RoHS (manufactured by KWB) with a measurement accuracy of up to 0.02 mm and a measurement speed of 1.5 m/s, powered by 1.45 V battery. Linear dimensions and diameter were recorded in millimeters. Statistical analysis was performed using IBM SPSS Statistics for Windows, Version 27 (IBM Corp, Armonk, New York).

## 3. Results

Unilateral variations were identified in three of the male cadavers (18.8%). The frequency of variations was 9.4% based on the total number of the dissected BPs. No statistically significant differences were observed in relation to sex or side.

**Figure 1:**
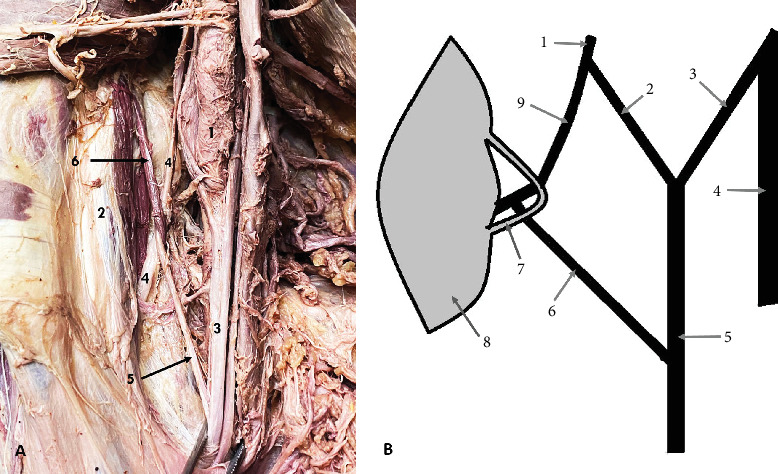
Communicating branch (CB) between the musculocutaneous nerve (MCN) and the median nerve (MN). (A) Cadaveric photo: 1—axillary artery; 2—coracobrachialis muscle (CBM); 3—MN; 4—MCN; 5—CB; 6—muscular band of CBM wrapped around the CB. (B) Diagram of the variation: 1—lateral cord; 2—lateral root of MN; 3—medial root of MN; 4—ulnar nerve; 5—MN; 6—CB; 7—muscular band of CBM wrapped around the CB; 8—CBM; 9—MCN.

The first variation was observed on the right side of a male cadaver, aged 79. It was presented as a communicating branch (CB) between the MCN and the MN. The MCN originated from the LC, and it pierced the CBM. Eighteen millimeters distal to the origin of the MCN, it gave off a CB to the MN, which originated within the CBM. Notably, the CB was encircled by a muscular band of the CBM (Figure 1). The length of the CB was measured at 27 mm. Distal to the origin of the CB, the MCN followed its usual course, providing branches to biceps brachii muscle (BBM) and brachialis muscle (BM), and terminated as the lateral cutaneous nerve of the forearm (LCNFA).

**Figure 2:**
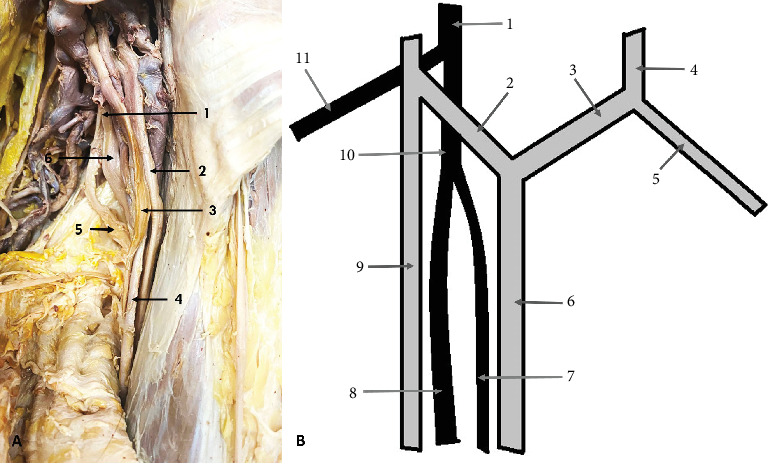
High division of the radial nerve (RN). (A) Cadaveric photo: 1—bifurcated RN; 2—median nerve (MN); 3—ulnar nerve (UN); 4—axillary artery; 5—medial division (MD); 6—lateral division (LD). (B) Diagram of the variation: 1—posterior cord; 2—medial root of MN; 3—lateral root of MN; 4—lateral cord; 5—musculocutaneous nerve; 6—MN; 7—LD of RN; 8—MD of RN; 9—UN; 11—RN; 12—axillary nerve.

The second variation observed was a high bifurcation of the RN in the axillary fossa into medial division (MD) and lateral division (LD), occurring 23 mm distal to the formation of the nerve. This variation was found on the left side of a male cadaver, aged 70. The LD of the nerve continued as the main RN, entering the anatomical space located immediately inferior to the axilla, known as the “triangular interval.” The MD traveled to the triceps muscle (TrM), where it divided into branches that supplied the muscle (Figure 2).

**Figure 3:**
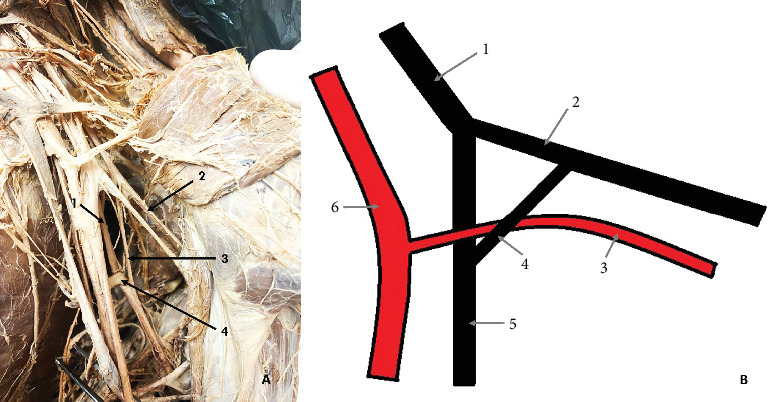
Communicating branch (CB) between the axillary nerve (AN) and the radial nerve (RN). (A) Cadaveric photo: 1—RN; 2—AN; 3—CB; 4—subscapular artery (SSA). (B) Diagram of the variation: 1—posterior cord; 2—AN; 3—SSA; 4—CB; 5—RN; 6—axillary artery.

The final variation was observed on the left side of a male cadaver, aged 40. The RN passed posterior and medial to the subscapular artery (SSA), where it received a CB from the AN. The CB measured 36 mm in length and was located anterior and lateral to the SSA. Together, the RN and the CB formed a loop beneath the SSA (Figure 3).

## 4. Discussion

Variations of the BP account for over 50% of neural deviations, as reported by Hassan and Jan [14], with the prevalence of BP variability ranging between 13.3% and 90% according to the literature [2, 3, 8, 14, 20, 23–29]. In our study, the incidence of BP variations was 18.8%, which aligns with the range reported in the literature.

Among the nerves of the BP, the MCN is one of the most variable, with its most frequent variation being the presence of CBs between the MCN and the MN [5, 16, 30–33]. Usually, the CB originates from the MCN and extends to the MN. Some authors suggest that fibers from the LC, which contribute to the MN, initially travel with the MCN and later rejoin to the MN via the CB [11, 15, 31, 34]. Shruthi et al. [12] proposed that the presence of CBs between these nerves might be attributed to variations in vascular development.

Numerous authors have proposed classifications for the variations of the MCN and the CBs between the MCN and the MN [6, 30, 31, 35–39]. Among these, Choi et al. [6] categorized the CBs based on their number into three patterns: Pattern 1—fusion of the MCN and the MN (19.2%); Pattern 2—a single CB between the MCN and the MN (72.6%), which is further subdivided into Pattern 2a—a single CB (69.9%) that included cases with the CB located proximal to the CBM (29.4%), from within the muscle (2%), distal to the muscle (54.9%) and instances when the MCN did not pierce the CBM (13.7%), and Pattern 2b—two or three branches from the MCN, forming a single CB to the MN (2.7%); Pattern 3—two CBs between the MCN and MN (6.8%). Loukas and Aqueelah [31] classified the CBs into four types: Type 1—CBs proximal to the entry point of the MCN into the CBM (45%); Type 2—CBs distal to the MCN entry point into the CBM, originating from within the muscle (35%); Type 3—the MCN does not enter the CBM (9%); and Type 4—a combination of the other types (10.5%). Uysal et al. [39] studied the MCN variations in fetuses and categorized the CBs into three types: Type A—CB located in the superior part of the arm (21.4%), Type B—CB located in the middle or distal part of the arm (64.3%), and Type C—CB originating from within the CBM (14.3%). The CB between the MCN and the MN identified in our study corresponds to Pattern 2a in the classification of Choi et al. [6], Type 2 in Loukas and Aqueelah classification [31], and Type C in Uysal et al. classification [39].

The reported incidence of CBs between the MCN and the MN varies significantly, ranging from 5.0% to 86.2% [6, 10–12, 19, 30–32, 36, 40, 41]. Maeda et al. [38] observed a significantly higher incidence of the CBs in specimens exhibiting additional heads of BBM. In the present study, we identified only one CB between the MCN and the MN, corresponding to incidence of 3.14%. This finding is notably lower than the incidence reported in the literature and can be explained with the small sample size of plexuses dissected and/or ethnic and biological characteristics of the studied population. Additionally, no excessive heads of the BBM were observed in our specimens.

Although CBs originating from the MN and traveling to the MCN have been reported in the literature at a low frequency [2, 3, 7, 33, 34, 38], we did not observe such variations in this study.

Most studies have reported that a single CB between the MCN and the MN is the predominant pattern [6, 7, 11, 12, 30, 31, 34, 37, 38], although two CBs have also been observed in some cases [6, 32, 33, 37, 42, 43]. Regarding the position of the CB relative to the entry point of the MCN into the CBM, some authors identified a predominance of the CBs located proximal to that entry point [31, 37, 39], while others have found distal CBs to be more frequent [6, 11, 30, 34]. In their review, Sirico et al. [16] categorized the CBs into five groups and found that the majority of CBs (45.97%) were located distal to the CBM.

Only limited number of studies [6, 31, 34, 36, 39] have described CBs originating from within the CBM as observed in our case. The reported frequency of this variation ranges from 2% to 14.3% [6, 34, 36, 39]. Loukas and Aqueelah [31] reported that 35% of the CBs between the MCN and MN were distal to the entry point of the MCN into the CBM and originated from within the CBM, which contrasts with the lower frequencies cited in other studies.

The variation in our study was found unilaterally in a male cadaver, consistent with previous reports indicating a predominance of unilateral CBs [6, 30, 31, 33, 34, 39, 40, 42, 44]. The CB we identified originated from within the CBM and, upon exiting the muscle, it was encircled by a thin muscular band. We could not find a similar variation in the published literature, which could be attributed to the lack of accurate descriptions of such variations and insufficient photographic documentation in previously published studies. This highlights the need for more comprehensive reporting and imaging to improve the understanding and classifications of BP variations.

According to its origin, the RN is considered the least variable among the terminal branches of the BP [5]. Variations of the RN are rare, with only a few studies reporting deviations from its typical pattern. In one cadaver, we observed that the RN divided in the axillary fossa into two divisions: medial and lateral. The MD supplied the TrM, while the LD continued as the main RN. Several authors have documented high divisions of the RN into two divisions with both divisions typically entering the radial groove. In these cases, one of the divisions continues as the main RN, while the other gives off all muscular and sensory branches of the nerve in the arm [2, 45–47]. The incidence of this variation ranges from 1.67% to 44% [2, 46, 48]. Raghavendra et al. [48] studied 50 upper limbs, including six fetal specimens, and reported a high division of the RN in 44% of the cases. They found that the axillary fossa was the more common site of division (77.3%) compared to the triangular interval (22.7%). The divisions of the RN were anterior and posterior in 59.1% of the cases and medial and lateral in 40.9%. In all specimens, the anterior or lateral divisions continued as the main RN. However, the authors did not specify whether the other division (posterior or medial) consistently supplied the TrM or whether both divisions entered the radial groove. Churikov et al. [46] described a high division of the RN into MD and LD in 11 cases (27.5%), terming this variant “double-trunk variant.” In their study, the MD did not give branches in the upper and middle third of the arm, while the LD provided muscular and sensory branches to the posterior arm muscles and the skin of the posterior arm and forearm. Both trunks entered the radial groove. In the case report of Ramasamy and Kalaivanan [49], the RN also formed two branches—anterior and posterior. The anterior branch continued as the main RN, innervating the extensor muscles of the forearm, while the posterior branch supplied the TrM. In our study, the LD entered the triangular interval, while the MD supplied the TrM without entering the interval. This variation closely resembles the pattern described by Ramasamy and Kalaivanan [49].

Variations in the branching pattern of the AN are infrequently reported in the literature, with the most common variation being the formation of common trunks with the lower subscapular and TDNs [5, 23]. In our study, we observed a CB between the AN and the RN, with the CB and the RN forming a loop beneath the SSA. Koizumi [50] studied 602 upper limbs and identified 7 CBs from the AN to the RN, all of which joined the RN on the posterior surface of the arm. Other authors have described variations where the SSA is encircled by two roots of the PC or RN, but no CBs between the AN and the RN were observed [51–53]. In a study on 100 upper limbs, Darji et al. [24] reported a CB between the AN and the RN in the axilla, but the SSA did not show any close relationship with the nerve structures. R. Rai and A.R. Rai [54] described a case where a CB between the AN and the RN was present but the CB crossed anteriorly the SSA, pierced the aponeurosis of latissimus dorsi, and joined the RN. Although these previously reported variations bear some resemblance to the one identified in our study, they differ significantly from the variation found by us. To the best of our knowledge, the variation we observed, involving a CB between the AN and RN forming a loop beneath the SSA, has not been described in the literature to date. Our findings highlight the need for further anatomical studies to document and understand such rare variations.

The presence of anatomical deviations in the terminal branches of the BP and its branches can result in unusual symptoms and atypical clinical manifestations, as well as nerve palsy or vascular complications due to altered relationships with surrounding muscles and vessels [18, 55]. Such variations complicate the diagnostic process and surgical procedures, increasing the likelihood of diagnostic and surgical errors [56]. Failure to properly assess BP variations increases the risk of iatrogenic injuries of the nerves or their variants during reconstructive procedures involving the BP or its branches, treatment of the humeral fractures, mobilization of the coracoid process, or upper extremity regional anesthesia, resulting in therapeutic failures [11, 15–17].

The MCN is susceptible to injury during traumatic events such as humeral fractures, stab or blunt wounds, and traction or stretching of the nerve. It is also at risk during surgical interventions, including shoulder surgeries or flap procedures following mastectomy, particularly when the CBM may be injured [11, 37, 57]. Although rare, cases of MCN entrapment have been reported, resulting from hypertrophy of the CBM or compression of the nerve due to fibrous bands between the BBM and the BM, mass lesions, or osteochondromas [7, 37]. Understanding variations of the MCN is crucial for diagnosing peripheral nerve lesions, performing neurophysiological examinations, and planning procedures such as shoulder reconstruction surgeries, shoulder arthroscopy, and the treatment of humeral fractures via the anterior approach. Such deviations are also significant in managing spasticity of the elbow flexor muscles, restoring elbow flexion through nerve transfers, and achieving effective infraclavicular nerve blocks [10–12, 16].

The presence of CBs between the MCN and MN must always be considered during clinical evaluations of nerve injuries, neuromuscular flap surgeries, peripheral nerve repairs, and exploratory interventions. It is also crucial for understanding the anatomy involved in anterior shoulder trauma repairs, regional anesthesia, and the functional impairments of the MCN and the MN [6, 11, 12, 30, 34, 58]. The presence of a CB can complicate the clinical presentation of MCN or MN lesions, leading to unexpected symptoms and potential misdiagnosis [19]. Lesions or compression of the MCN proximal to a CB can manifest as weakness of the forearm flexors and thenar muscles [7, 11, 12, 33, 37, 58], as well as conditions such as carpal tunnel syndrome, pronator teres syndrome, anterior interosseous nerve syndrome, or MN neuropathy in the hand. These manifestations depend on the specific nerve fibers contained within the CB [7, 30, 34]. Proximal CBs are often closely associated with the AA, and this relationship can lead to arterial compression, resulting in ischemic pain or arterial insufficiency during shoulder joint movements [11, 37]. Surgical damage to a CB may result in postsurgical complications, including MN deficits [30].

The CB between the MCN and the MN identified in this study is at substantial risk of injury due to entrapment syndromes of the MCN, lesions of the CBM, or surgical procedures. Due to the close relationships between the CB connecting the AN and the RN observed in our study and the SSA, the artery may be compressed by the branch, potentially leading to reduced blood supply and ischemic changes [11, 37, 53]. This anatomical variation may also predispose the vessel to injury during anesthetic block procedures or bypass surgeries involving the subclavian artery and the AA [53]. This highlights the importance of recognizing and preserving such variations to minimize complications.

The RN is particularly vulnerable in cases of humeral shaft fractures, compression by intermuscular septum or fracture callus, as well as during various surgical procedures [48, 59]. The presence of two trunks of the RN can further complicate the diagnostic process, treatment, and postoperative management of the RN injuries [46, 48]. Such a variation may predispose to failure in reconstructive surgeries of the nerve. If one of the trunks is not identified and repaired during surgery, persistent neurological deficits may result in the postsurgical period [46].

The variations of the BP are of high clinical importance. Profound knowledge of such deviations is essential to avoid misdiagnosis of clinical symptoms and to prevent nerve injuries during a variety of procedures. Deviations in the terminal branches of the BP may necessitate changes in treatment plans and present new opportunities for nerve transfers, enhancing surgical outcomes.

## 5. Conclusion

This study on the BP demonstrated that the variations in the terminal branches of the PC are more prevalent than those of the LC in the Bulgarian population. Notably, we identified two previously undescribed variations: a CB between the MCN and the MN, enclosed by a muscular band of the CBM, and a CB between the AN and the RN, forming a loop around the SSA. While these findings contribute to the understanding of BP variability, our study is limited by the small sample size. Further investigations with larger cadaveric populations are required to confirm and expand upon these results. Understanding these variations is essential for improving diagnostic accuracy and minimizing surgical complications.

## Data Availability

We confirm that the data supporting the findings of this study are available within the article.
